# A deep learning framework for bone fragment classification in owl pellets using YOLOv12

**DOI:** 10.1038/s41598-025-15906-9

**Published:** 2025-08-13

**Authors:** Nik Fadzly, Lay Wai Kean, Siti Nuramaliati Prijono, Rini Rachmatika, Siti Zulaika, Mohd Nasir, Hasber Salim

**Affiliations:** 1 School of Biological Sciences, Universiti Sains Malaysia, USM, Pulau Pinang, 11800 Malaysia; 2 Applied Zoology Research Center, National Research and Innovation Agency (BRIN), Jalan Raya Bogor KM 46, Cibinong, Kec. Cibinong, Kabupaten Bogor, Jawa Barat, 16911 Jakarta, Indonesia; 3 Pusat Penyelidikan Arkeologi Global, Universiti Sains Malaysia, Pulau Pinang, 11800 Malaysia

**Keywords:** Artificial intelligence, Owl pellets, Bone classification, Machine learning, Object detection, Machine learning, Animal physiology

## Abstract

Non-invasive monitoring of small mammal populations is critical for both biodiversity conservation and integrated pest management, particularly in agroecosystems. Barn owl (*Tyto alba*) pellet analysis has long served as a valuable tool for inferring prey abundance, yet conventional bone classification is labour-intensive and requires specialized expertise. Here, we introduce a deep learning framework that automates the detection and classification of rodent bone fragments from owl pellets using the YOLOv12 object detection architecture. A dataset comprising 978 annotated images, encompassing skull, femur, mandible, and pubis bones, was used to train and validate the model, achieving high detection performance (precision = 0.90, recall = 0.90, mAP@0.5 = 0.984, F1-score = 0.97). The model demonstrated strong generalization across samples from Malaysia and Indonesia. We further developed a Python-based inference script to estimate rodent abundance using skull and paired bone counts. This AI-assisted workflow reduces human error, increases processing throughput, and enables scalable rodent monitoring. By enhancing ecological inference from pellet studies, our approach supports timely biodiversity assessments and pest surveillance strategies across diverse landscapes.

## Introduction

Accurate monitoring of small mammal populations is vital for ecological research, biodiversity conservation, and integrated pest management, particularly in agricultural landscapes where rodent infestations cause significant crop losses. Barn owls (*Tyto alba*) have long been recognized as effective biological control agents, preying on rodent species such as *Rattus argentiventer* and *Rattus norvegicus* in rice fields, oil palm plantations, and urban environments^1–4^. Owl pellets which are regurgitated masses of indigestible material including bones, teeth, and fur, offer a non-invasive method for assessing prey composition and estimating rodent abundance in each habitat^5–7^. Pellet collection is considered a non-invasive method because it involves gathering naturally regurgitated materials from known roosting or nesting sites, without any physical interaction, handling, or disturbance to the owls themselves. This passive sampling technique avoids direct trapping, tagging, or manipulation of animals, thus minimizing stress and behavioural alteration.

Despite their ecological value, conventional pellet analysis remains a laborious process that requires osteological expertise and considerable manual effort. The classification of skeletal fragments is often hindered by variability in observer skill, degradation of samples, and lack of standardization^8,9^. These limitations reduce throughput and introduce inconsistencies, posing challenges for large-scale ecological monitoring and timely pest control responses. While distinguishing major anatomical elements may seem straightforward, human classification is subject to variability in anatomical knowledge, visual fatigue, and subjective judgment, particularly when fragments are degraded or incomplete. Deep learning offers standardized, repeatable classifications and can process large datasets rapidly without retraining or fatigue, thereby reducing inter-observer bias and scaling up ecological assessments.

Recent developments in artificial intelligence (AI), particularly in computer vision and deep learning, present opportunities to automate ecological data workflows. Convolutional neural networks (CNNs), including the You Only Look Once (YOLO) object detection models, have demonstrated high accuracy and efficiency in real-time classification tasks across ecological domains such as camera-trap image analysis, animal identification, and acoustic monitoring^10–12^. However, their application to anatomical fragment classification in owl pellets remains largely unexplored^13^.

This study aims to address this gap by developing and validating a YOLOv12-based deep learning model to automatically detect and classify rodent bones in barn owl pellets. The model was trained on 978 annotated images representing four key anatomical categories: skull, femur, mandible, and pubis. A complementary Python-based inference pipeline was developed to estimate rodent abundance using skull and paired bone counts. This AI-assisted framework offers a scalable, reproducible, and non-invasive approach for small mammal monitoring, enhancing ecological inference in both conservation and pest management contexts.

## Results

**Fig. 1:**
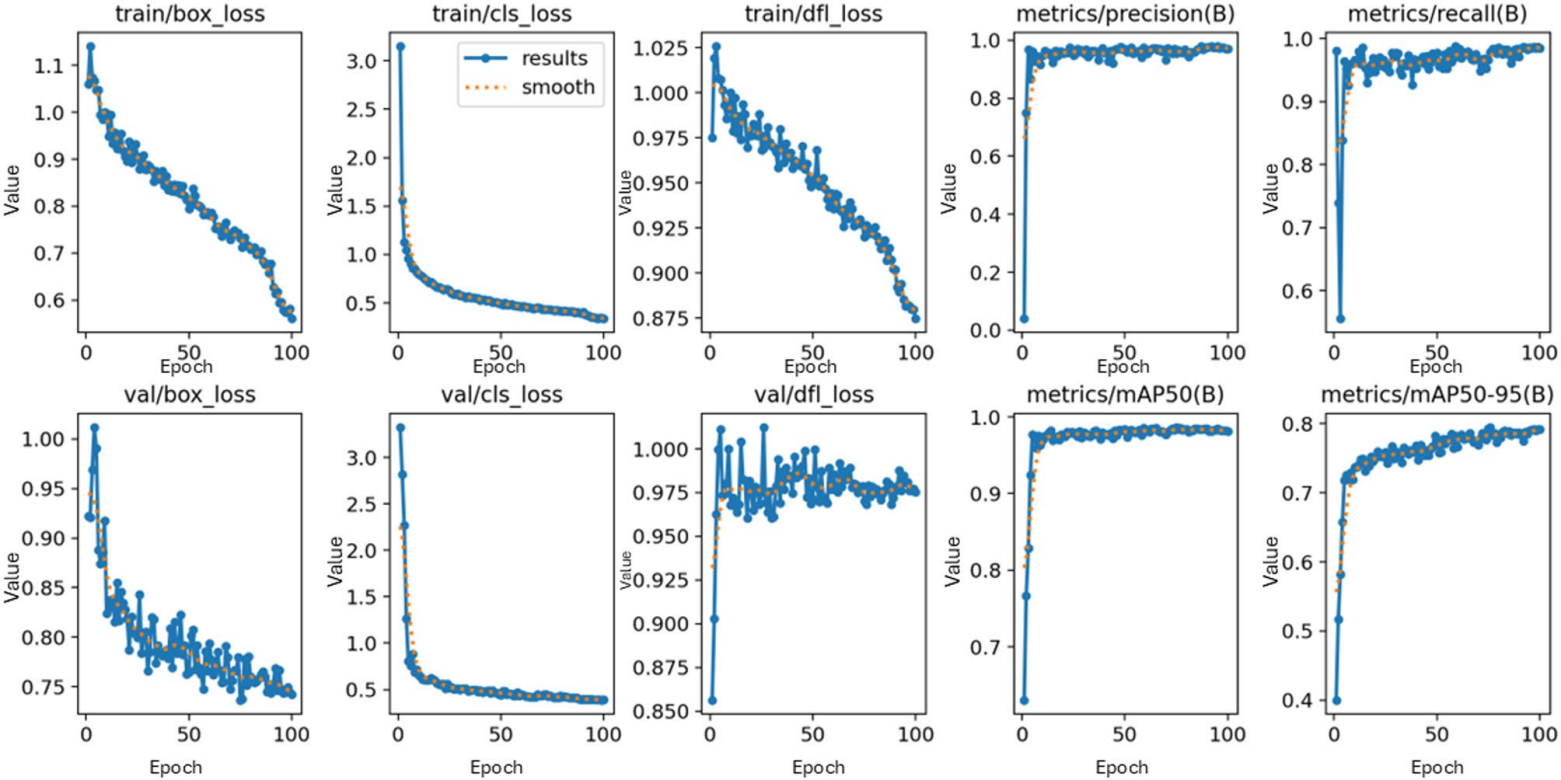
Training loss components and model performance metrics over 100 epochs. The plots include bounding box loss, classification loss, distribution focal loss (DFL), precision, recall, mAP@0.5, and mAP@0.5:0.95. Both raw and smoothed values are shown.

The training performance of the YOLOv12 model is summarized in Fig. 1, which shows the evolution of training and validation metrics over 100 epochs. Training losses including box loss, classification loss (cls_loss), and distribution focal loss (dfl_loss) continued to decline across epochs, with a noticeable acceleration after epoch 80. Validation losses did not follow identical patterns: dfl_loss fluctuated around a stable average, while box and cls losses gradually decreased. Precision and recall increased sharply and reached stable values above 0.90 by approximately epoch 5, indicating early convergence in classification performance.

However, we recognize that precision and recall alone do not adequately reflect object detection quality, as they primarily assess classification and not background discrimination or localization accuracy. Although the model did not exhibit signs of overfitting, the sustained reduction in training losses and variable validation behaviour suggest that it may not have fully converged and could be underfittted. This underfitting may be attributed to the relatively small training dataset and the limited number of training epochs, which may have constrained the model’s ability to optimize fully.

**Fig. 2:**
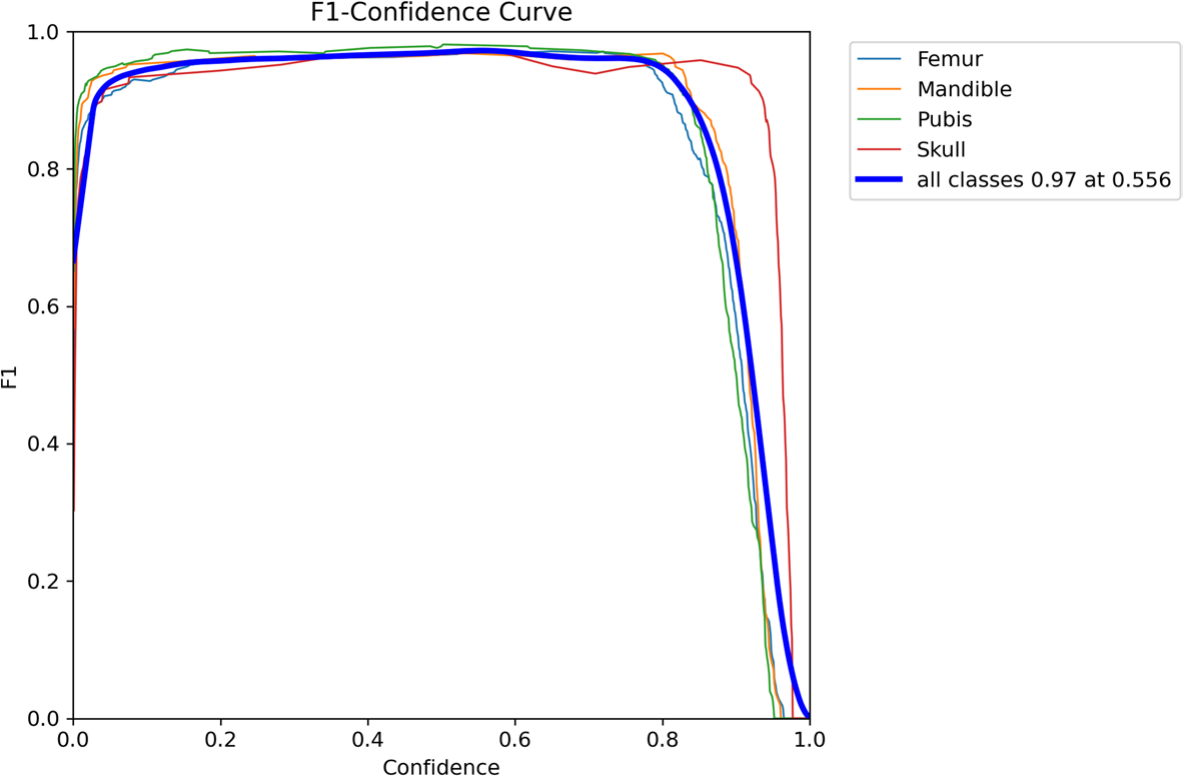
F1 score across varying confidence thresholds for each bone class, including the average F1 performance. This curve aids in selecting an optimal confidence threshold for prediction.

Figure 2 illustrates the F1-confidence curve across multiple thresholds. The F1-score, which represents the harmonic mean of precision and recall, reached its peak value (F1 = 0.97) at a confidence threshold of 0.666. This value was selected as the optimal trade-off point for model deployment, balancing sensitivity and specificity in detecting bone fragments. Adjusting the confidence threshold enables tailoring the model’s performance to various operational requirements. For example, prioritizing high recall for biodiversity assessments or high precision for pest surveillance.

To provide a clearer breakdown of model performance, we present per-class precision, recall, F1-score, and the number of ground-truth instances (support) for each anatomical category (Table 1). These metrics complement the confusion matrix by quantifying the model’s classification consistency and detection quality across classes.

**Table 1:** F1-score was computed as the harmonic mean of precision and recall. ‘All’ represents macro-averaged metrics across the four bone classes. Background is not explicitly labelled in YOLOv12 outputs but is reflected in false positives across classes, particularly as quantified in the confusion matrix.

Class	Support (Instances)	Precision (*P*)	Recall (*R*)	F1-Score	mAP@0.5	mAP@0.5:0.95
Femur	210	0.962	0.967	0.964	0.973	0.731
Mandible	110	0.972	0.953	0.962	0.981	0.801
Pubis	190	0.979	0.974	0.976	0.994	0.810
Skull	49	0.959	0.954	0.956	0.981	0.871
All	559	0.968	0.962	0.965	0.982	0.803

**Fig. 3:**
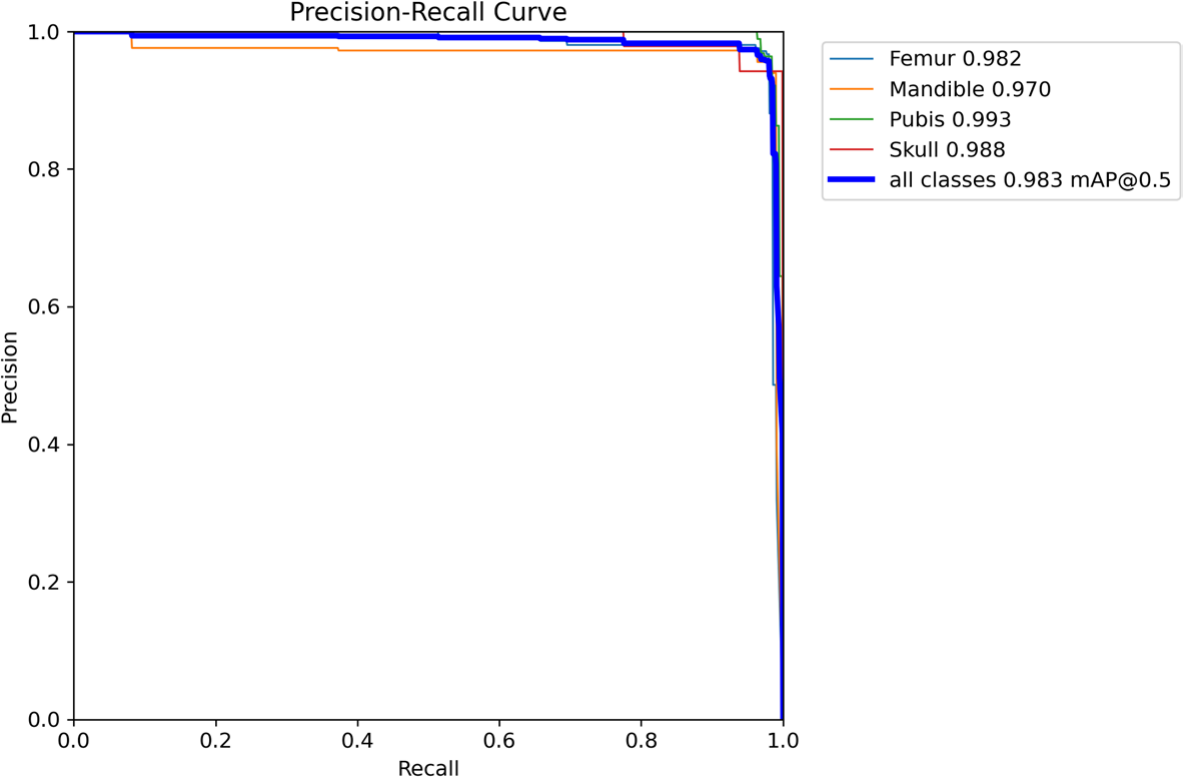
Precision–Recall curves for each class, visualizing model performance across recall thresholds. These plots help to assess class-specific precision trade-offs.

The model’s detection performance was further evaluated using mean Average Precision (mAP) at different Intersection over Union (IoU) thresholds. The model achieved an mAP@0.5 of 0.982 (Fig. 3), indicating that 98.2% of predictions had at least 50% overlap with ground-truth bounding boxes. The mAP@0.5:0.95, a stricter metric that averages detection accuracy across IoU thresholds from 0.5 to 0.95 in 0.05 increments, was 0.803. This measure reflects the model’s robustness in detecting small and fragmented anatomical structures under varying localization precision requirements.

**Fig. 4:**
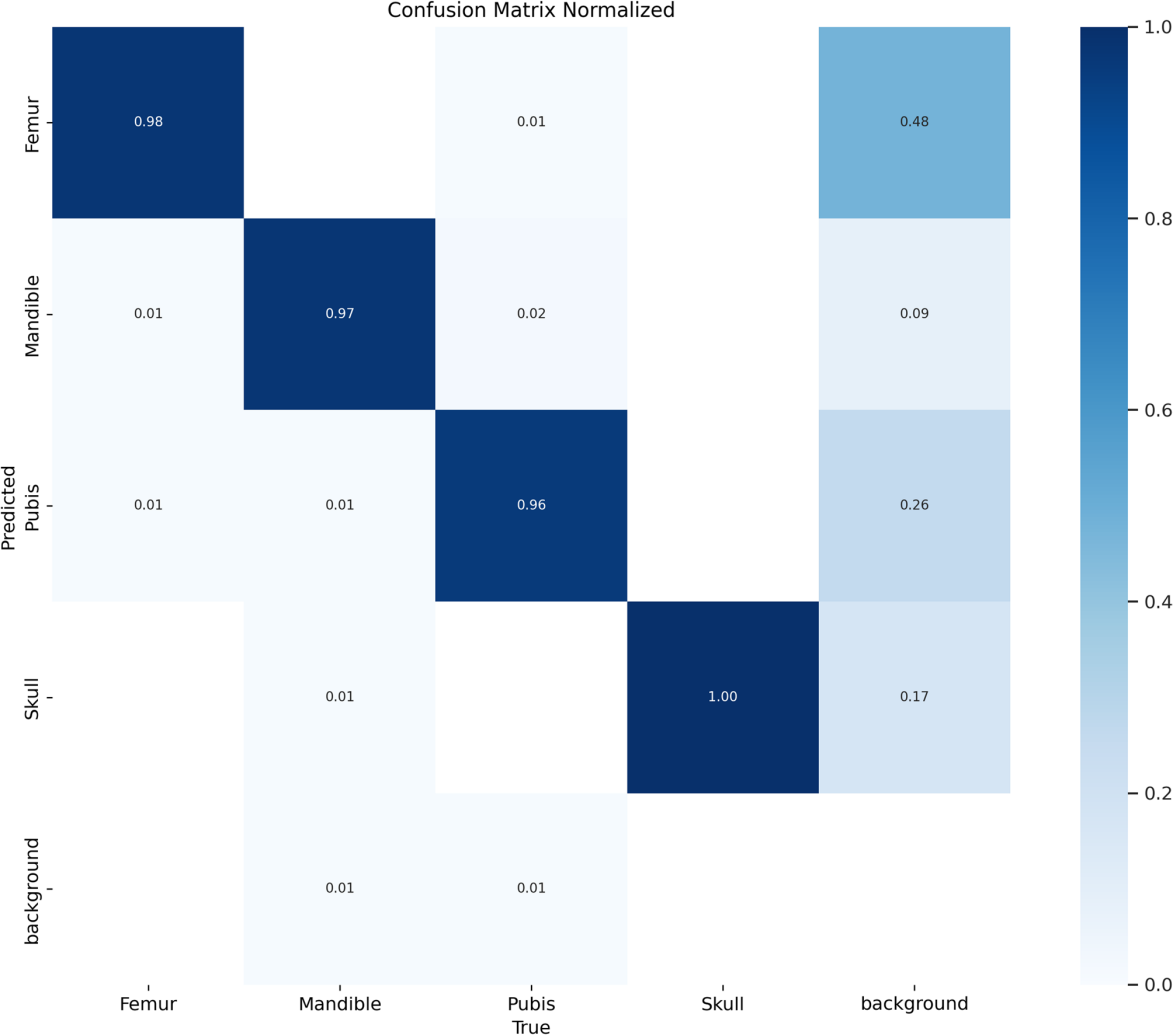
Normalized confusion matrix showing classification performance across all bone classes. Values are normalized by the true class (row-wise) to highlight class-specific accuracy.

Classification accuracy across anatomical categories is shown in the normalized confusion matrix (Fig. 4). The model correctly classified femurs (98%), mandibles (97%), pubis bones (96%), and skulls (100%) with minimal confusion between these classes. However, a high rate of false positives was observed in the femur class, where 48% of true background instances were misclassified as femurs. This suggests the model occasionally confuses background debris or shadows with anatomical features, particularly for small objects resembling femur contours. The ‘background’ class refers to all image regions that do not contain target anatomical structures (i.e., skull, femur, mandible, pubis). These include pellet debris, fur, unidentifiable fragments, and empty regions in the image. During annotation and training, such regions were implicitly modelled by the YOLOv12 objectness loss function, which learns to distinguish between foreground (annotated bones) and background (non-annotated areas). No explicit label was assigned to background regions, but the model treats any area not enclosed by a bounding box as background during learning.

**Fig. 5:**
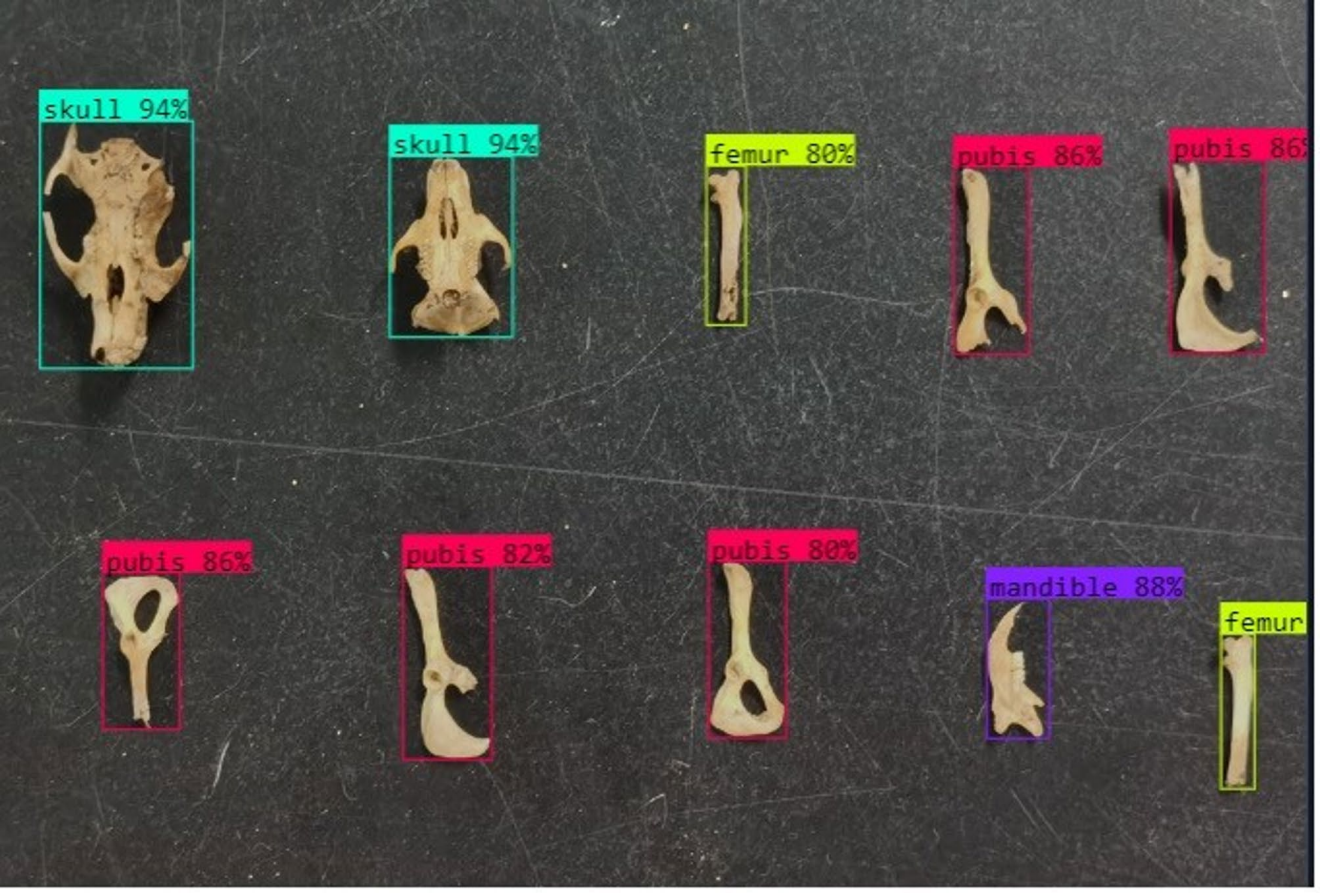
Example output from the model detection on a validation data.

The trained model was integrated into a Python-based inference script for downstream application. Using a high-confidence threshold of 0.8, the model produced clear bounding boxes and class labels for all bone types (Fig. 5). The script supports both individual and batch image analysis. Individual skull detections were used as primary indicators of minimum rodent abundance, while paired bones (e.g., femur, mandible, pubis) provided secondary estimates when skulls were absent or damaged. Population estimates were computed based on the number of non-overlapping bone detections, with skulls used as the most conservative metric. Maximum abundance estimates considered surplus paired bones relative to known skeletal composition in *Rattus* species.

**Fig. 6:**
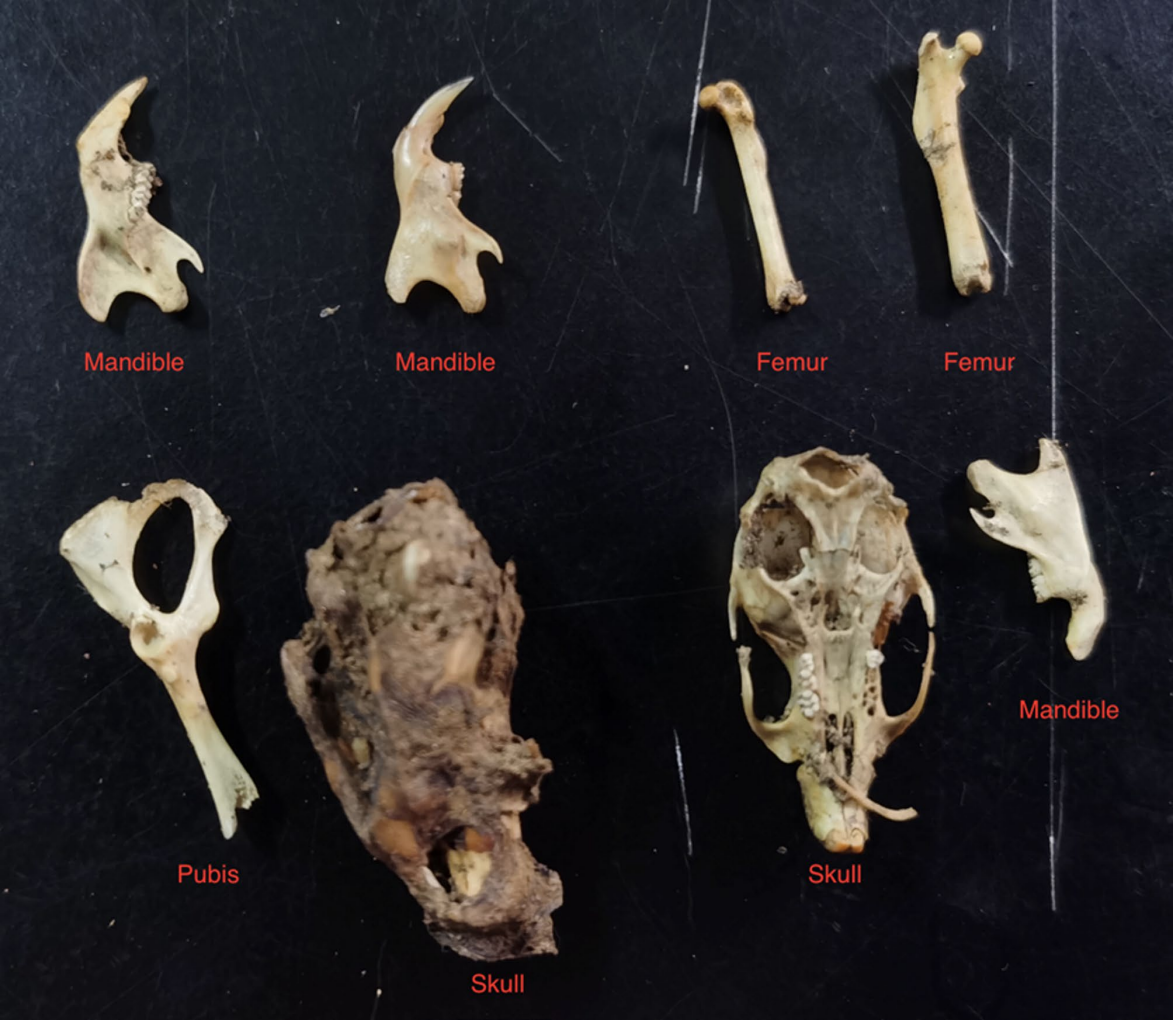
Example of the type of bones extracted from an owl’s pellet.

Figure 6 presents an example inference output, highlighting the model’s capacity to distinguish anatomical elements under variable lighting, orientation, and background conditions. The script also includes aggregation functions for batch processing, enabling automated estimation of rodent populations from large image datasets.

## Discussion

This study demonstrates the feasibility and effectiveness of using deep learning to automate bone fragment classification from Barn Owl (*Tyto javanica javanica*) pellets. To our knowledge, this is the first application of an object detection framework specifically trained to recognize skeletal fragments in owl pellets for ecological monitoring. Conventional pellet analysis remains dependent on manual osteological examination^6,7,13^, limiting throughput and increasing susceptibility to human error. Our YOLOv12-based model overcomes these limitations by offering rapid, reproducible, and scalable detection of rodent skeletal components, directly supporting both conservation and pest management efforts. Although sample preparation remains a prerequisite for both manual and AI-based methods, the classification step itself is significantly accelerated with deep learning. Once deployed, the model can analyze hundreds of fragments within seconds, outperforming human observers in throughput and consistency, especially when dealing with large-scale pellet surveys. For example, a researcher would only need to take pictures of the bones during different sampling times throughout the year, and our Python script can provide the counts separated by sampling times within less than 5 min. There would be no further need to collect and keep the bone specimens.

The model achieved strong performance across multiple metrics, including high mAP@0.5 (0.984), precision (0.90), and F1-score (0.97), comparable to or exceeding other ecological deep learning applications^11,14^. These results indicate that constrained classification tasks such as distinguishing four anatomically distinct bone types may yield more consistent outcomes than broader species recognition models, which often report lower accuracy due to variability in morphology and environmental conditions^15^. The relatively high accuracy in our study reflects the distinct structural features of the selected bones (skull, femur, mandible, pubis), which were intentionally chosen for their recognasibility and frequency in owl pellets^16,17^.

The confusion matrix revealed consistent misclassification of background regions as femurs, with 48% of true background instances predicted as femur. This suggests the model occasionally confuses debris or shadows with anatomical features, particularly when background textures resemble long bone structures. It is possible that this confusion may be linked to underfitting of the objectness component during training. As YOLOv12 incorporates an objectness loss term, future training iterations could explore tuning its relative weight to improve background discrimination.

Detecting small, occluded, or degraded bone fragments remains a known challenge in ecological imagery^10,18^. Although the model performed well with well-preserved fragments, digestion-related degradation, overlapping structures, and poor contrast likely reduced sensitivity for bones under 1 cm in length. Nevertheless, the model successfully identified complex anatomical features such as the ischial ramus and pubic tubercle, indicating a capacity to manage biologically noisy and visually variable data^19^. A limitation of this study is the relatively small test set (81 images), which may contribute to variation in performance metrics. While this split provided sufficient object-level annotations (~ 200 bones), it may not fully capture the variability present in natural owl pellet samples. Future studies should consider implementing K-fold cross-validation or increasing the test split to 20% to improve robustness and reduce performance variance across different subsets.

Our dual strategy for abundance estimation by using skull counts as a conservative metric and paired bones as secondary indicators shows enhanced reliability, particularly when skulls were absent or damaged. This method is consistent with prior pellet analysis protocols^5,7^, and supports ecological inference with minimal disturbance to wildlife, in contrast to live trapping or invasive sampling. These advantages are especially relevant in protected areas or large-scale agricultural systems, where non-invasive monitoring is preferred.

Being able to automatically estimate rodent abundance from owl pellet-remains holds substantial ecological and applied value. Rodents are among the most prolific agricultural pests globally, responsible for significant yield losses in rice, oil palm, and other crops^20^. Accurate and timely rodent population estimates are crucial for deploying targeted pest management interventions before outbreaks occur. Conventional trapping methods are costly, time-consuming, and often logistically infeasible at landscape scales. By contrast, pellet-based monitoring offers a cost-effective, passive, and non-invasive alternative that reflects long-term prey presence. Automating this process through AI-driven image analysis enhances scalability and responsiveness, enabling continuous surveillance and real-time integration into integrated pest management (IPM) frameworks. This approach also supports ecological studies by providing high-resolution data on small mammal community composition and predator–prey dynamics, particularly in biodiversity-sensitive areas.

Despite the model’s efficiency, certain structural limitations of YOLO-based detectors warrant discussion. The use of bounding boxes restricts the model’s ability to handle overlapping or fragmented objects, a common scenario in densely packed pellet contents. It should be noted that effective image-based detection required physical disaggregation of the pellet to expose bone fragments, as intact pellets occlude skeletal elements. Emerging segmentation models such as Mask R-CNN or YOLOv5-seg offer improved spatial precision through pixel-level object delineation^21–23^ and may be better suited for resolving ambiguities when skeletal elements are visually merged. Future adaptations of this pipeline could incorporate instance segmentation to address these challenges.

Although our model performed consistently on samples from two countries, broader testing across different owl populations, seasons, and prey assemblages is required to validate robustness under diverse field conditions. Environmental heterogeneity and taxonomic variability are known to affect model performance^9,10^. Approaches such as domain adaptation, ensemble learning, or synthetic data augmentation^24^ may further enhance cross-habitat applicability.

This study also shows the value of cloud-based platforms like Roboflow and Google Colab in democratizing access to deep learning tools. However, practical constraints such as computational limits, session timeouts, and reliance on internet connectivity; highlight the need for lightweight, locally deployable solutions. Beyond pellet analysis, this approach may be extended to other ecological use cases involving fragmented biological material, such as scat analysis, archaeological assemblages, or forensic bone classification. With minimal modification, our model and inference pipeline can be adapted to identify additional skeletal elements or distinguish between species. Such versatility supports a wide range of conservation, agricultural, and research applications where accurate species monitoring is critical.

Our results demonstrates that deep learning, specifically the YOLOv12 object detection framework can be effectively used to automate the classification of rodent bones in barn owl pellets. The high precision, recall, and mean Average Precision values achieved shows the robustness and reliability of our approach, even under biologically and visually complex conditions. By integrating this model with an inference pipeline capable of estimating rodent abundance, we offer a scalable, non-invasive tool for ecological monitoring.

Future research should focus on expanding anatomical class diversity, improving sensitivity for smaller bone fragments, and incorporating segmentation models to address overlapping object challenges. Additionally, enhancing model generalizability across habitats and prey profiles will be critical for broader deployment. The open accessibility of our model and dataset encourages reproducibility and adoption by other researchers, contributing to the growing integration of artificial intelligence in conservation biology and ecological informatics.

## Methods

### Study sites and sample collection

Barn owl (*Tyto javanica javanica*) pellets were collected from two locations: Bumbung Lima (Penang, Malaysia) and the Animalium facility at the National Research and Innovation Agency (BRIN), Bogor, Indonesia. The Malaysian site host active barn owl populations integrated into rodent pest management programs, whereas the Indonesian counterpart consists of birds kept in aviary for educational purposes^1,4^. Samples were collected between June and July 2024, with additional archived samples included to increase dataset size and validity. Pellets were retrieved from nest boxes and the vicinity areas around the supporting poles, using gloved hands and stored in individually labelled plastic bags. Owl handling was non-invasive and approved by the Animal Ethics Committee, Universiti Sains Malaysia (Exemption ID: USM/IACUC/2025/[EXEMPTION][0009]). No owls were physically touched or harmed during this experiment. The sample collection was approved by the Barn Owl Research (BORG), USM and Badan Riset Indonesia (BRIN).

### Pellet processing and bone extraction

In the laboratory, pellets were soaked in distilled water to soften the matrix. Bone fragments were separated using sterilized forceps and rinsed in 70% ethanol for disinfection. Bones were sorted by type and stored with location and date metadata.

### Image acquisition and annotation

Bones were photographed individually or in groups on a uniform matte background using a Huawei Mate 30 Pro 5G smartphone mounted 50 cm above the sample. Images were captured under ambient light with varied orientations to increase diversity. Manual annotations were performed using Roboflow, assigning bounding boxes to four anatomical categories: skull, mandible, femur, and pubis. A second reviewer audited 15% of annotations for consistency.

### Dataset augmentation

The initial dataset consisted of 228 high-resolution raw images of dissected owl pellets collected between June and July 2024. These images were then augmented using Roboflow to enhance model generalization. Augmentation techniques included horizontal and vertical flipping, grayscale conversion (15%), and contrast variation. The final dataset comprised 978 enhanced images with 2,644 annotated bone fragments (femur: 1,087; pubis: 816; mandible: 493; skull: 248). This augmented dataset was used for training and evaluation. The images were split into training (858), validation (39), and test (81) sets.

### Model training

Model development used YOLOv12 implemented via the Ultralytics framework in Google Colab. The network was trained for 100 epochs with a batch size of 16 and input resolution of 640 × 640 pixels. Data augmentation included random flip, mosaic, and brightness adjustment. The model was evaluated using precision, recall, mAP@0.5, and mAP@0.5–0.95 metrics. A confidence threshold of 0.8 was selected based on F1 optimization.

### Inference and abundance estimation

Inference was performed using a custom Python script. Bone fragments were detected from new images, filtered by confidence score, and counted by class. Skull counts were used as primary indicators of minimum rodent abundance. Paired bone types (e.g., femur, pubis, mandible) were used as supplementary indicators in the absence of skulls. The script supports both single-image and batch analysis.

Rodent abundance was estimated using both minimum and maximum thresholds derived from bone detection counts. The minimum estimate corresponded to the number of detected skulls, assuming each skull represented one individual. In the absence of skulls, the minimum was inferred from the number of paired post-cranial bones (e.g., two femur or two mandibles = one individual). The maximum estimate was calculated by summing the total number of surplus bones (e.g., mandibles, femur, pubis) divided by the number expected per individual, providing a conservative upper bound of rodent presence in the sample. This approach is consistent with methods in pellet-based mammal community assessments^5^.

### Model testing and validation

Generalizability was tested using pellet images not included in the training set, selected from the same annotated dataset collected in June–July 2024 in both Malaysia and Indonesia. The trained model was applied to these previously unseen images for rodent population estimation based on detected bone types.

### Use of generative AI and AI-assisted technologies in the writing process

During the preparation of this manuscript, the authors used generative AI tools (ChatGPT, OpenAI, version GPT-4, SciSpace and Grammarly) to assist with improving the clarity, conciseness, and formatting of the text, as well as reorganizing content to match journal-specific structure. No AI tool was used to generate original scientific content, results, or interpretations. All outputs from the AI tool were reviewed, edited, and verified for accuracy by the authors. The authors take full responsibility for the content of the manuscript.

## Data Availability

All datasets, trained models, and inference scripts used in this study are publicly available. The annotated dataset can be accessed via Roboflow: [https://universe.roboflow.com/ml-usm/updated-rat-bone-v2/dataset/6](https:/universe.roboflow.com/ml-usm/updated-rat-bone-v2/dataset/6). The trained YOLOv12 model and Python inference notebook are available on GitHub: [https://github.com/nroselnik/Counting-ratbones-from-owl-pukes](https:/github.com/nroselnik/Counting-ratbones-from-owl-pukes). A permanent archival copy of the dataset is hosted at OSF: [http://doi.org/10.17605/OSF.IO/XDM4G](http:/doi.org/10.17605/OSF.IO/XDM4G).
